# Primary and Secondary Physiological Stress Responses of European Sea Bass (*Dicentrarchus labrax)* Due to Rearing Practices under Aquaculture Farming Conditions in M’diq Bay, Moroccan Mediterranean: The Case of Sampling Operation for Size and Weight Measurement

**DOI:** 10.3390/life13010110

**Published:** 2022-12-30

**Authors:** Soumaya Cheyadmi, Housni Chadli, Hassan Nhhala, Bouchra El Yamlahi, Mohammed El Maadoudi, Ayoub Kounnoun, Francesco Cacciola, Ayoub Ez-Zaaim, Hicham Chairi

**Affiliations:** 1 Research Team in Biological Engineering, Agri-Food and Aquaculture, Department of Biology, Polydisciplinary Faculty of Larache, Abdelmalek Essaâdi University, Tetouan 93030, Morocco; 2 Aqua M’diq Marine Fish Farm, M’diq 93200, Morocco; 3 Department of Earth Sciences, Faculty of Sciences and Technologies of Tangier, Abdelmalek Essaâdi University, Tetouan 93030, Morocco; 4 Laboratory of Biomedical Genomics and Oncogenetics, Faculty of Sciences and Technologies of Tangier, Abdelmalek Essaâdi University, Tetouan 93030, Morocco; 5 Regional Laboratory for Analysis and Research—National Office for Food Safety ONSSA, Tangier 90000, Morocco; 6 Department of Biomedical, Dental, Morphological and Functional Imaging Sciences, University of Messina, 98125 Messina, Italy

**Keywords:** stress, sampling for weight-length measurement, European sea bass, M’diq Bay, physiological responses

## Abstract

Globally, aquaculture contributes to sustainable food and nutritional stability. However, stress conditions constitute a major threat affecting farmed-fish welfare and fish farms’ performances. In this regard, the present study was aimed at measuring and comparing in-situ (offshore) the physiological stress responses caused by recurrent sampling operations for length–weight measurement. Studied fish were European sea bass *Dicentrarchus labrax* sub-adults and adults reared in intensive farming conditions in M’diq Bay, on the Moroccan Mediterranean coast. The physiological stress response was evaluated by measuring blood biomarkers, including cortisol, glucose, lactate, total cholesterol and total proteins levels, and hematocrit percentage. The hypercortisolemia, hyperglycemia, hyperlactatemia and hypoproteinemia detected in the post-sampling state in both age groups of *D. labrax* indicated stress tendencies and a high sensitivity to aquaculture practice-related stress factors, with major and minor responses detected in the same age and same stress conditions. It is also interesting to note that the fish age and the time-course of the sampling operation had a statistically significant effect in terms of the physiological response (with *p* < 0.01 and *p* < 0.001, respectively). In conclusion, the present study showed that sea bass subjected to various stressful situations in intensive farming conditions displayed a physiological stress response specific to its age, to the individual status, as well as to the duration of stressor.

## 1. Introduction

The aquaculture sector, typically marine fish-farming, has become an important key contributor to sustainable food and nutritional stability, and is also a lever for the development of marine coastal clusters. Currently, this aquaculture activity is the fastest growing aquatic-food production source in the world (5%/year) [1]. However, despite the phenomenal growth of its production, aquaculture is experiencing various problems, including variability in productivity and profitability. Indeed, marine fish are sensitive animals and their survival in captivity is full of challenges to overcome, especially towards stress [2], and one of the causes limiting fish farm profitability is losses caused by stressed conditions. Understanding the relationship between the expansion of industrial fish-farming and fish-welfare science has become increasingly important in order to provide optimal survival conditions for fish and management systems [3]. Recently, fish farmers have become aware of the ethical needs of fish, namely their health, positive emotions, growth and survival. They have moved from the concept of aquaculture being that of hunting to that of care [3], taking full responsibility for respecting and ensuring the five freedoms of fish welfare, which positively influence their economic interests [4]. For this purpose, sufficient skills, equipment and technologies, in addition to the spirit of scientific research are required [3].

The European sea bass *Dicentrarchus labrax* represents, in economic terms, the main species of intensive industrial marine fish-farming in Morocco. In fact, the uncontrolled phenomenon of stress and its various factors constitute a major threat for farmed fish and for aquaculture producers. This stress can affect the acceptance, profitability, efficiency, quality and quantity of the aquaculture product and sector in general due to the massive mortality and harmful economic losses [4].

Physiologically, the experience of one or more intra- and interspecific stress-inducing factor(s) by the fish leads to unexpected synergistic or antagonistic cascading effects via allostatic mechanisms, beginning with activation of the Hypothalamic–Pituitary–Interrenal (HPI) axis, followed by a complex series of neurological, biochemical, energetic, physiological and behavioral adjustments to maintain their internal homeostasis and resistance to death [2,5,6,7,8].

Quantification of the three stress responses—Primary R. = alarm phase; Secondary R. = resistance phase; Tertiary R. = phase of compensation or death—requires the measurement of the levels of their indicators in pre- and post-stress states: namely, the level of stress hormones, metabolites and mineral salts; enzyme activity and gene expression; change in swimming and feeding behaviors; the monitoring of zoo-sanitary indicators and zootechnical performances [2,6]. These indicators could be assessed from a wide choice of samples (blood, tissue homogenates, rearing water, etc.) using successful and highly developed methodologies [6,8]. It is also important to take into consideration the relationship between the nature of the stressor(s) and the appropriate biomarkers to be measured during a stressful event [6].

Cortisol is the main glucocorticoid hormone determining the state of fish welfare; particularly reflecting the primary response caused by acute stress [8], its release by interrenal cells [9] is regulated by a negative feedback controlled by the HPI axis [10]. Once glucocorticoids circulate, they are associated with a suite of secondary responses that help fish to flight, fight and cope with the threatening challenges by stimulating several aspects of the control of aerobic and anaerobic metabolism of glucose, lactate, total protein, triglycerides and total cholesterol [2,6,11]. Glucose and lactate can provide information on the aerobic and anaerobic mobilization of energetic resources to several fish tissues respectively, and the swimming and muscular capacities of fish to respond to the stressor [2,12]. Meanwhile, the blood nutritional indicators of total protein, total cholesterol and triglycerides can provide information on recent dietary history and current levels of mobilized energy stores in farmed fish [6]. Generally, sea bass and stressed fish are characterized by a decrease in the level of these metabolites in a post-stress state; the opposite case is seen for glucose and lactate, which increase under the effect of stress [2], with the exception of a few stressful conditions that lead to metabolic dysfunction and depression [13,14] in which the evolution of these post-stress indicators is reversed. Leucocyte and hematological profiles, including blood cell counts and hematocrit percentages, are the indicators of the health and immune status of fish [15,16].

The purpose of the present study was to assess the presence of physiological stress responses specific to fish age and status caused by aquaculture operations in farmed fish in intensive farming conditions. It aimed to measure and to compare in-situ (offshore) the primary and secondary physiological stress responses, induced by sampling operations for weight and length measurement, in subadult and adult European sea bass farmed in floating cages in M’diq Bay on the Moroccan Mediterranean coast. The blood puncture was performed on forty-eight fishes (12 each by age and by stress state). Cortisol, glucose, lactate, total cholesterol and total protein levels and hematocrit percentages were used as physiological biomarkers. In addition, this study was carried out in order to study the possibilities of mitigating the impacts of aquaculture practices on the welfare, growth and survival performances of farmed fish, and the sustainability of the sea bass fish-farming sector, especially in terms of the biosecurity concept and stress management.

## 2. Materials and Methods

### 2.1. Ethical Procedures

The present study was performed according to the guidelines of the European Union Council (2010/63EU) dedicated to the protection of animals during handling and scientific experiments in the laboratory [17]. To minimize the suffering of the animals and their stress, the duration of pulling the fish out of the sea during fishing and the morphometric measurements was reduced (4 s < 15 s) and the blood puncture was undertaken for 3 to 5 min per fish.

### 2.2. Biological Material and Study Site

**Figure 1:**
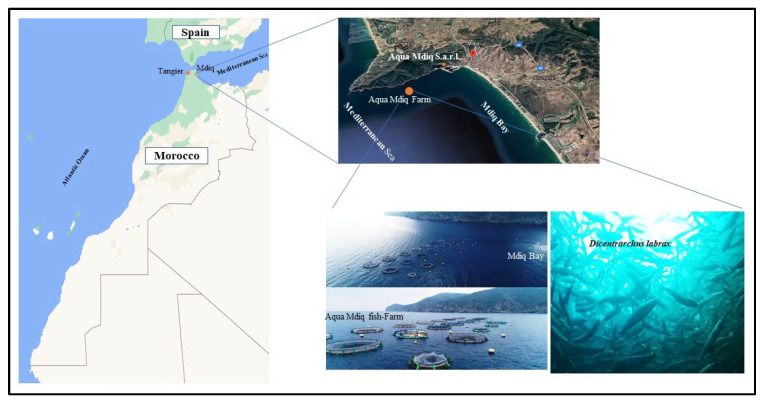
The geographical position of the Aqua M’diq farm at M’diq Bay, located one nautical mile from the port of M’diq, Morocco.

Our study was carried out on 48 fish of European sea bass in intensive farming conditions, (24 fish per age group; 12 fish presenting each stress state). Two groups of fish were studied; the first group having had a one year stay and the second group having had two years’ stay at the same fish farm. The fish were caught randomly on the day of sampling operation from two different floating circular cages at the Aqua M’diq fish farm, located in the M’diq Bay on the Moroccan Mediterranean coast (Figure 1). The characteristics of the farm site have been accurately described by recent studies [18,19].

The studied fish were healthy and did not show any signs of weakness. For the chosen size, we visually tried to choose individuals of approximately the same size in each age group, and those with sizes different to the designated range were returned to the tanks. The physico–chemical parameters of the study site were measured using a HANNA multiparameter probe (mode HI98196, Hanna Instruments Morocco, Agadir, Morocco). The water in the tank was renewed during transport and the physico–chemical parameters were also measured in order to ensure the stabilization of the water temperature during transport from the farm to the port.

### 2.3. Stress Factor: Sampling Operations

Sampling operations were carried out for the monthly monitoring of zootechnical parameters and to assess the general condition of the stock and their performance, in particular, growth. This operation is considered to be a stress-inducing factor since it involves the temporary removal of the fish from its environment. The sampling duration varies from 40 min to 1 h 30 min depending on the number of cages (1 to 3), and was performed according to the following steps:

- Fasting the fish 24 h before this operation;
- Crowding the fish in another net on the cage surface to facilitate their fishing;
- Transferring the caught fish in tanks filled with well-oxygenated seawater (4 s per scoop net);
- Transporting the tanks by a speedboat to the port (less than one nautical mile away = 13 min);
- Anesthesia of the fish in another bath;
- Realization of morphometric measurements, namely weight and length;
- Recovery of the fish in the wake-up tanks; and
- Re-transportation of fish and transferring them to their own cages.

### 2.4. Physiological Quantification of Stress 

The quantification of stress due to the sampling operation in European sea bass was carried out in two stress states in 12 fish per state for each age group:

- Pre-stress state: blood was drawn from 12 fish immediately after their catch from their rearing cages;
- Post-stress state: blood was drawn from 12 fish before their return to their own cages.

### 2.5. Experimental Study

This study was conducted at the end of April 2021, with the blood sampling from the two life stages in two stress states undertaken on the speedboat offshore in the M’diq Bay. The fish were caught randomly and then anesthetized with clove oil (0.5 to 1 mL/L) in order to minimize the blood sampling stress. Next, they were put back in ice to prevent them from drying out. The fish were caudally venipunctured using a sterile pediatric syringe (21 G; 2.5 mL). The blood was subsequently stored in heparinized pediatric tubes (4 mL) and the tubes were placed in a cooler at 4 °C. Subsequently, the morphometric parameters (weight and length) of each fish were measured using an ichthyometer and a balance (accuracy 10 kg. 0.1 g), respectively. Organosomatic indices were also measured using a 0.01 g precision balance and calculated by the following Equations [6]:

$$\mathrm{Viscerasomatic}\;\mathrm{Index}\;(\%):\;\mathrm{VSI}\;=\frac{\mathrm{Fish}\;\mathrm{Viscera}\;\mathrm{weight}\;}{\mathrm{Body}\;\mathrm{weight}}\times 100$$

$$\mathrm{Spleen}\;\mathrm{somatic}\;\mathrm{Index}\;(\%):\;\mathrm{SSI}\;=\frac{\mathrm{Weight}\;\mathrm{of}\;\mathrm{spleen}\;}{\mathrm{Body}\;\mathrm{weight}}\times 100$$

$$\mathrm{Hepatosomatic}\;\mathrm{Index}\;(\%):\;\mathrm{HSI}\;=\frac{\mathrm{Weight}\;\mathrm{of}\;\mathrm{liver}\;}{\mathrm{Body}\;\mathrm{weight}}\times 100$$

$$\mathrm{Intestine}\;\mathrm{somatic}\;\mathrm{Index}\;(\%):\;\;\mathrm{ISI}\;=\frac{\mathrm{Weight}\;\mathrm{of}\;\mathrm{intestine}\;}{\mathrm{Body}\;\mathrm{weight}}\times 100$$

$$\mathrm{Gonadosomatic}\;\mathrm{Index}\;(\%):\;\mathrm{GSI}\;=\frac{\mathrm{Weight}\;\mathrm{of}\;\mathrm{gonad}\;}{\mathrm{Body}\;\mathrm{weight}}\times 100$$

### 2.6. Physiological Stress Indicators Studied

The hematological indicators, including those chosen in the present study—namely cortisolemia, glycemia, lactatemia, cholesterolemia, proteinemia, and hematocrit percentage—are indicative of the general health of the fish and their physiological, metabolic and immune status [16].

Upon arrival at Aqua M’diq society (M’diq port), three drops of fresh blood were used to measure blood glucose, total cholesterol and lactate levels using portable readers and specific strips (BeneCHEK TM Plus Multi-Monitoring System, Parapharmacy Morocco (from USA); and THE EDGE TM Blood Lactate monitoring system, EBM ELEVAGE BIOTECH MAROC (from China)) [6] according to the manufacturers’ instructions, then the concentrations were converted from mg/dl to mmol/L. The blood was centrifuged (1250× *g* for 15 min; Sigma centrifuge) and a drop of plasma was used to measure the concentration of total plasma proteins using an optical portable refractometer (Brix at 20 °C, BK-PR32, Educomptoir society in Morocco (from China)) [20]. The percentage of blood hematocrits was then measured by the following Equation (6)

$$\;\frac{\;\mathrm{Volume}\;\mathrm{of}\;\mathrm{red}\;\mathrm{blood}\;\mathrm{cells}}{\mathrm{Total}\;\mathrm{blood}\;\mathrm{volume}}\times 100\;$$

The obtained plasma was recovered in Eppendorf tubes and stored at −20 °C for subsequent cortisol analyses which took place in the regional laboratory of ONSSA, Tangier. The quantitative determination of endogenous concentrations of plasma cortisol in sea bass *D. labrax* was carried out using a fish cortisol ELISA–Competitive Kit (Cusabio Biotech Co., Ltd., Allemande, Houston, TX, USA) [21]. The optical density of each well (Blank, Standards, and Fish Plasmas) was measured at 450 nm at least for 5 min using a microplate spectrophotometer (Elx 808, Biotek company, Winooski, VT, USA) connected to the computer. Plate layout was created using Gen5 and Expert curve software to record optical densities in order to calculate standards and target sample concentrations.

### 2.7. Statistical Analysis

The obtained data were statistically analyzed by IBM SPSS Statistic (Version-25) with a significance risk α = 0.05. The values of the morphometric parameters, the organosomatic indices and the physiological biomarkers of stress were expressed in the form: Mean ± Standard deviation (Min~Max).

The physiological stress indicators were statistically confirmed by the Shapiro–Wilk normality test and by the non-parametric tests (UMann–Whitney and Kruskal–Wallis). Levene’s test was performed to test the homogeneity of variance hypothesis. Two-way multivariate ANOVA (two-way ANOVA), Student’s *t*-test, Bonferroni’s post-hoc correction, Between-Subject Effects test were used to compare and study the main effects and influence of sampling operations on farmed *D. labrax* stress responses between different life stages as well the states of stress. Finally, Principal Component Analysis was used to reduce the data in order to recognize the global physiological effects of sampling operations in the two life stages in pre- and post-stress states.

## 3. Results

### 3.1. The Morphometric Parameters of European Sea Bass and the Physico–Chemical Parameters of Fish Farming

Our study was conducted with the aim of studying and comparing the primary and secondary physiological stress responses of two life stages of the European sea bass *D. labrax* induced by the common aquaculture operation, “sampling operation for length- and weight-measurement”, in intensive farming conditions in the M’diq Bay. The age and morphometric description of our biological material are presented in Table 1 and Table 2.

During this study, the physico-chemical parameters of seawater in and around the floating cages of the Aqua Mdiq fish farm were as follows: temperature (17.4~18 °C), pH (7.7~8.26) and dissolved oxygen saturation percentage (88~91%). During the round-trip transport, the temperature and pH of the water in the tank filled by live fish were the same as those of sea water.

### 3.2. The Physiological Stress Response Due to the Sampling Operation in Farmed European Sea Bass

- **Cortisolemia (ng/mL):**

The obtained results (Table 3) showed, a statistically very highly significant increase in mean cortisolemia in the post-stress state (*p* < 0.001) in subadult (+56%) and adult (+42%) sea bass compared to that recorded in the pre-stress state. Adults presented significantly higher values (187.34 ng/mL ± 46.20 and 320.70 ng/mL ± 72.66) compared to subadults (115.74 ng/mL ± 22.32 and 264.54 ng/mL ± 50.34) in pre–and post-sampling states, respectively (with *p* < 0.001; age category).

- **Glycemia (mmol/L):**

The average blood glycemia recorded in the two life stages of farmed *D. labrax* in the post-sampling state was very significantly higher (*p* < 0.001), in the order of 48% for subadults (9.53 mmol/L ± 3.25) and of 46% for adults (14.05 mmol/L ± 4.67), compared to that recorded in the pre-sampling state (4.99 mmol/L ± 1.93 in subadults and 7.60 mmol/L ± 3.20 in adults). Adult fish also showed significantly higher blood glucose concentrations compared to subadults (*p* < 0.01) (Table 3).

- **Lactatemia (mmol/L):**

The mean blood lactatemia found in the post-sampling state in subadult sea bass (13.61 mmol/L ± 3.56) and adults (17.65 ± 6.83 mmol/L) was significantly higher (*p* < 0.001) than that found in the pre-sampling state (9.12 mmol/L ± 4.55 in subadults and 10.35 mmol/L ± 5.38 in adults). The concentrations recorded in large fish were higher, however, this increase is not statistically significant (*p* > 0.05) for the “Age” category (Table 3).

- **Cholesterolemia (mmol/L):**

Total cholesterol levels found in farmed *D. labrax* blood did not show any significant variation in terms of age and stress status categories (*p* > 0.05). The subadults revealed a slight decrease of around 0.244 mmol/L (−4%; *p* > 0.05) in the post-sampling state compared to the pre-stress state. In contrast, adults showed a slight increase in this blood indicator of around 0.63 mmol/L (+16%; *p* > 0.05) in the post-sampling operation compared to the pre-sampling state (Table 3).

- **Proteinemia (g/dL):**

Mean plasmatic proteinemia was significantly lower in the post-sampling condition (*p* < 0.001; Stress state category) in subadult and adult *D. labrax* (*p* < 0.05; age category). This hypoproteinemia is around 1.28 g/L (−20%; *p* < 0.05) for subadult fish and around 1.19 g/dL (−18%; *p* < 0.001) for adult fish (Table 3).

- **Hematocrit percentage (%):**

Subadult fish did not showed any difference and variation in terms of the average of hematocrit percentage in both states, it is around 41.00%. Indeed, adult fish revealed a significant decrease in the percentage of hematocrit from 54.35% in the pre-stress state to 47.60% in the post-stress state (−22%; *p* < 0.05) (Table 3)

### 3.3. Statistical Analysis of the Obtained Data

The Shapiro–Wilk Normality test showed that the average of evaluated concentrations of the blood markers follow the Normal law (*p* > 0.05) according to the difference between the stress states and the age of *D. labrax*, with the exception of the means of blood glucose and total cholesterol recorded in the pre-sampling state in subadults as well as the mean of proteinemia found in adults in the pre-stress state (*p* < 0.05). These results are also confirmed by QQ plot and by Henry’s line.

The statistical distribution of the obtained concentrations according to the “State of stress” and “Age” categories was provided by the non-parametric U-Mann–Whitney and Kruskal–Wallis tests, by two-way ANOVA, by the Levene and Student *t*-tests, and by Bonferroni’s post-hoc correction (Table 3). They statistically showed the very highly significant increase (*p* < 0.001) in the mean values of cortisolemia, glycemia and lactatemia and the very highly significant decrease (*p* < 0.001) in total protein levels in subadult and adult European sea bass after the sampling operation. The Between-Subjects Effects Test (two-way ANOVA) of our present study showed, in general, that the categories “Age” and “Stress state” had a statistically highly significant effect on the blood indicators of European sea bass with *p* < 0.01 for “age” and *p* < 0.001 for “stress state” categories. In contrast, the interaction between “Age and Stress State” had no statistically significant effect (*p* > 0.05) on the distribution of blood indicators of stress in farmed sea bass.

**Figure 2:**
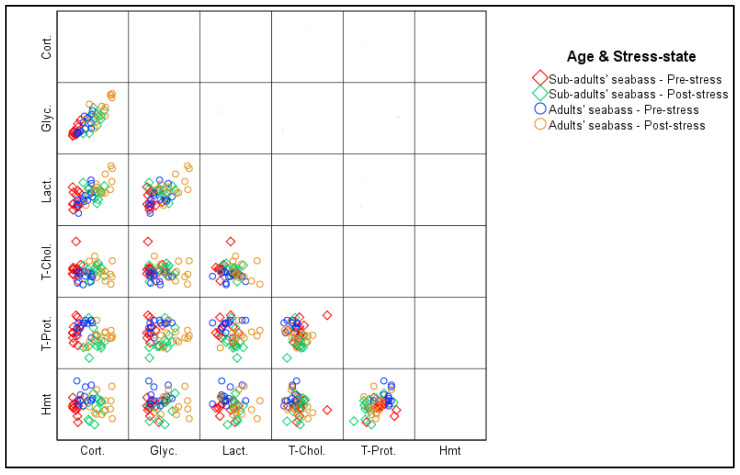
Linear correlation matrix between the evolution of physiological stress biomarkers in sea bass individuals (N = 48) according to age and the stress states during the sampling operation at M’diq Bay (Cort.—Cortisolemia; Glyc. —Glycemia; Lact. —Lactatemia; T.Chol. —Total Cholesterol; T.Prot. —Total Proteins; Hmt. —% Hematocrits).

The correlation test (Figure 2 and Table 4) shows the presence of a strong positive correlation, very highly significant (*p* < 0.001), between the evolution of the concentrations of plasma cortisol and blood glucose and lactate, as well as between the evolution of these two metabolites. It also showed the presence of a statistically significant negative correlation (*p* < 0.05) between the plasma cortisol and total protein levels. In addition, there was a highly significant (*p* < 0.01) positive correlation between total protein levels and hematocrit percentage.

**Figure 3:**
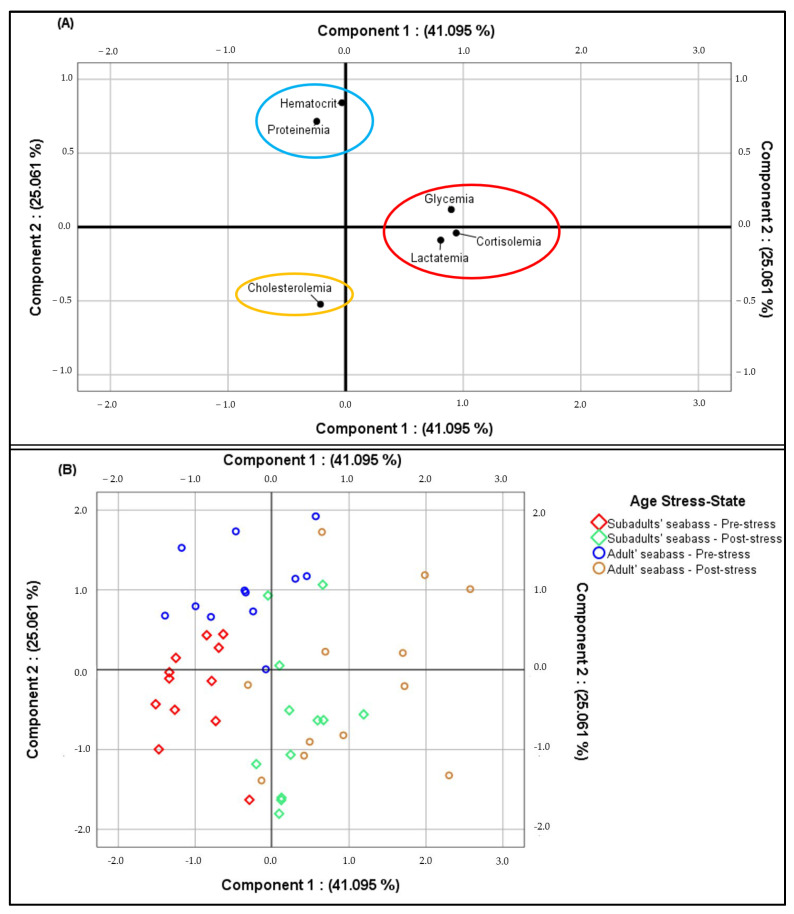
Principal component analysis representing the mean total (**A**) and individual concentrations (**B**) of blood indicators in subadult and adult *Dicentrarchus labrax* in pre- and post-stress states.

Principal component analysis (PCA) is feasible for our results, as demonstrated by the Kaiser-Meyer-Olkin Index (KMO = 0.59), by the Bartlett Sphericity Test (*p* < 0.001) and by two eigenvalues > 1. All the stress indicators, with the exception of total cholesterol level, have a good quality of the represented information (>50%). The PCA has established a stress panel related to the sampling operation for plasma cortisol, blood metabolites (glucose, lactate, total protein and cholesterol) and hematocrit percentage according to the fish age and the stress induction time (Figure 3A,B).

The PCA has also shown the occurrence of three distinct groups of physiological stress responses in the two life stages of farmed seabass stressed by the sampling operation, in which the blood biomarkers of each group have the same evolution and they are statistically positively correlated (Figure 3A and Table 4). Group 1 represents cortisol, glucose and lactate levels, which increased in the post-sampling state (Figure 3A,B and Table 3). Group 2 represents total protein levels and hematocrit percentage, which decreased in the post-sampling state (Figure 3A,B and Table 3), and Group 3 represents the total cholesterol level, which did not show any significant effect under the same stress conditions (Figure 3A,B and Table 3).

## 4. Discussion

Understanding the mechanisms underlying the physiological cascades in fish, which could be incriminated by acute or chronic stress related to aquaculture practices, is essential to ensure the survival of the farmed fish and the development and sustainability of the aquaculture industry. The change in certain primary and secondary physiological indicators of stress is particularly useful in marine fish farmed in floating cages for the purpose of evaluating the effects of stressful situations under farming conditions, in order to minimize and manage them. The present study highlights how the sea bass *Dicentrarchus labrax* physiologically responds to the recurrent aquaculture operation “Sampling Operation for length and weight measurement”.

For subadult fish aged 504 days (364 days of stay on the farm), mean weight 125.88 ± 21.33 g and mean size 22.83 ± 1.39 cm, the results showed a very highly significant hypercortisolemia (+56%; *p* < 0.001) and hyperglycemia (+48%; *p* < 0.001), significant hyperlactatemia (33%; *p* < 0.05), non-significant hypocholesterolemia (−4%; *p* > 0.05) and significant hypoproteinemia (−20%; *p* < 0.05) in the post-sampling state compared with the concentrations found in the pre-stress state. In contrast, no difference was revealed for the mean percentage of hematocrit in the pre- and post-sampling states.

Furthermore, the results found in adult fish aged 878 days (646 days of stay on the farm), average weight 338.88 ± 13.95 g and average size 31.4 ± 0.76 cm, showed also a very highly significant hypercortisolemia (+42%; *p* < 0.001), highly significant hyperglycemia (+46%; *p* < 0.01) and hyperlactatemia (+41%; *p* < 0.01), and very highly significant hypoproteinemia (−18%; *p* < 0.001) in the post-stress state. On the other hand, they showed a non-significant hypercholesterolemia (+16%; *p* > 0.05) and a significant decrease of around 22% (*p* < 0.05) in the percentage of hematocrits.

Based on these results, the response of the *D. labrax* subadult and adult to stress induced by this aquaculture operation followed the general stress response pattern [22] which is evidently translated by the significant increase in blood levels of cortisol, glucose and lactate as well as the significant decrease in protein levels. The reliability of these parameters makes it possible to identify them as useful biomarkers for the application of a standardized system for measuring stress due to aquaculture operations in this species.

In addition, the average concentrations of cortisol, glucose and lactate seem to be higher in both life stages in the pre-stress state. This could be due to the effect of the noise of the speedboat motor [23], the crowding of fish in the fishing net at the floating cage surface and their fishing by the dip net (15 min in total) as demonstrated by Marino et al. and by Di Marco et al., in adults (2 years; 371 ± 93.8 g; 28.2 ± 2.1 cm) and subadults (139.8 ± 8.0 g), respectively [24,25].

In terms of comparison between the two age groups, the blood values of certain indicators, namely cortisol, glucose, total protein levels and the hematocrit percentages appear very high in adult fish compared to subadults, for both pre-and post-sampling states (*p* < 0.01; Between Subject Effects Test), indicating the relative sensitivity of large European sea bass. Sopinka et al. mentioned that cortisol is a reliable biomarker to show the effects of age and body size in *D. labrax* subjected to stressful situations [6].

Indeed, we visually detected aggressive and audacious behavior in the majority of large fish during each operation step. However, a great disparity in the standard deviations of the indicators “Cortisol”, “Glucose” and “Lactate” was marked by the Levene test indicating the heterogeneity of the individual responses in the two life stages of *D. labrax*. This individual variability has also been marked in other studies carried out on this species but subjected to other various stressful conditions [24,26,27,28,29,30]. It could be argued that stressed fish with low concentrations of plasma cortisol and blood metabolites could increase the clearance rate of these biomarkers to manage and maintain the tolerable levels in bloodstream [2]. From these results, it is important to deduce the existence of age-specific stress responses as well as the individual status of European sea bass during the sampling operation. Our results agree with those showing the presence of a specific response to this sentinel species according to the time-course and intensity of the stressful situation, particularly in the variation of the level of the stress hormone (cortisol) and metabolites (glucose, lactate and total protein) [22].

The results obtained by Samaras et al. showed that the monthly re-exposure of the sea bass (once/month, for 4 months) to the same stressful event could cause a repeatability in terms of the cortisol response, with some presenting major responses and others presenting minor responses in a consistent and constant way, or else in a variable way [26]. Accordingly, each monthly repetition of the sampling operation could reinduce primary and secondary responses in the sea bass, with a difference in the degree of response between individuals depending on the weather, the biological conditions of the livestock and the farming environment.

The increased cortisol levels detected in the two life stages of *D. labrax* after the sampling operation could indicate that the aquaculture practice favors the activation of the HPI axis in *D. labrax* and all fish. This response will, in turn, activate the physiological, cerebral, osmotic and immune functions as well as modify the feeding and swimming (space utilization) behaviors of fish. Indeed, their over-secretion affects and modulates all these functions and disrupts the zoo-sanitary and zootechnical performances, thus depleting its immunocompetence and therefore beginning a trajectory leading to death [10,11,14,27,31,32].

The individual cortisolemias found after the operation confirms the results of several authors revealing that the sea bass at various stages of its life cycle—fry, juveniles, subadults and adults—is a species with very high sensitivity to various stressful situations [22,26,27,29,32,33] compared to other marine fish. The basal levels, recorded by all the dedicated studies on this species and cited in this paper, vary between 20~40 ng/mL and 100~300 ng/mL, and the post-stress levels range from 80~100 to 400~1000 ng/mL. This makes the sea bass an ideal biological model among marine fish to study the dynamics of cortisol hormone signaling [29].

Hypercortisolemia has also been reported in European sea bass subjected to various stressors related to aquaculture practices and the change of biotic and abiotic environmental parameters of the aquatic farm, such as: confinement at high stockage density (34.6 kg.m^−3^) accompanied or not by infectious infestation by *Vibrio anguillarum* (10^5^ CFU per fish) [34], short exposure to physico–chemical water quality changes (high TAN = 18 mg/L and hypoxia = 20% O_2_ saturation) [28], chasing (6 min) following the air exposure of fish (1.5 min) [22], confinement of fish (30 min every 2 days) with chasing (5 min every 2 days) and air exposure (1 min every 7 days) [32], exposure to sound and seismic waves (0 to 5000 Hz) using an air-gun blast in the open sea [5], the chronic variation in water temperature (increase and decrease; from 17 °C to 23 °C and vice versa) during farming [35], and exposure of fish acclimated to extreme salinities (3 and 30‰) to ectothermic stress (8 °C) [33].

The post-stress variation of the secondary indicators “Glucose”, “Lactate” and “Total Protein” shows the presence of a more acute response to stress [34]. The present study also showed that the sampling operation resulted in significant mean hyperglycemia and hyperlactatemia positively correlated with plasma cortisol levels in both age groups (R > 0.5; *p* < 0.001). This response is important in large fish, and could be attributed to the strong swimming behavior exhibited by adult fish in a limited environment, which does not allow them to escape from stressful events such as fishing with a dip net and confinement in a small reservoir of anesthesia.

Hyperglycemia reported in stressed fish, including *D. labrax*, resulting from the stimulation of glycogenolysis and gluconeogenesis by the stress hormones catecholamines and cortisol [36]. This response indicates the aerobic capacities exerted by the animal in order to provide them with the necessary energy to fight, flight, cope with and recover from the stressful event [13,32,37].

Relative hyperlactatemia also results from enhanced gluconeogenesis as an attempt to meet energy demands and maintain glucose levels [38], which could be confirmed by the positive correlation between these two metabolites (R > 0.5; *p* < 0.001). It indicates increased anaerobic metabolic activity due to active swimming and intensive muscular exercise typically associated with stressful conditions [14,37]. Schreck and Tort cited that this intense response could cause delayed mortality that could start from 30 min from the cessation of muscular activity due to the depression of the acid–base balance [2]. This hypothesis agrees with the farm’s observations where the fish mortality rate increased after each program of this aquaculture operation.

Protein levels were significantly reduced in the post-sampling state in both age groups, indicating their mobilization to meet the energy requirements necessary to maintain the increased physiological activity due to stress [39]. This reduction is negatively correlated with the level of cortisol (*p* < 0.05), and could be explained by the hypothesis that hypercortisolemia activates the catabolism of total proteins [13,37] following peripheral proteolysis [40]. The long-term consequences of stress and protein catabolism cause skeletal muscle breakdown, reduction and/or cessation of fish growth, immune dysfunction and thus predisposition of fish to death [13].

All of these studies have shown that the acute increase in cortisol, lactate and blood glucose levels, in addition to the acute decrease in total protein levels in the farmed *D. labrax* during its response in some stress cases could persist for a few h to a few days (4 h, or 24 h up to 72 h), and that the fish can recover their internal homeostasis and naturally express their swimming and feeding behaviors without affecting their survival rate.

With regards to the total cholesterol level, the sampling operation did not reveal any significant effect on this indicator. The results of our study agree with those found by Di Marco et al. in the sea bass *D. labrax* [25].

The decrease in the average percentage of hematocrits detected in adult sea bass in the post-sampling condition, which correlated with the decrease of total plasma proteins (*p* < 0.01), could be correlated with a decrease in hemoglobins and the number of red blood cells and with an increase in white blood cells [33,41]. This phenomenon can be explained by the effect of significant erythrophagocytosis following damage or shrinkage of red blood cells due to stress. This decrease could also indicate the susceptibility of adult fish to anemia [42] compared to subadult sea bass during the handling operation. This anemia could, in the long-term, damage fish hemoglobins and degrade their membrane proteins following a developed septicemia due to the production of reactive oxygen species by fish neutrophils, macrophages and endothelial cells [42].

The blood basal and post-stress concentrations of the 6 measured biomarkers of farmed sea bass in the present work vary with regard to those found by other studies, and also vary between individuals of the same or different age groups studied. Indeed, the degree of the severity of the stress response on the fish could be strongly attributed to the type of stress-inducing factor, its intensity and its duration, as well as to the difference in the study context (experimental tanks or in cage at sea) and the difference in the water physico–chemical quality, particularly the temperature and the salinity between the various farming areas. In addition to the fish status and its biological levels, other important factors include as age, zootechnical performance, behavior and coping styles, the health status and the ontogenesis of the individual (including the stressful experiences received during its survival), the tolerable capacity of the animal, the vertical transmission of stressful phenotypes, the degree of individual phenotypic plasticity, the robustness and variety of the epigenetic network linked to environmental changes, etc. [6,7,26,37,43].

## 5. Conclusions

The sampling operation during the rearing of seabass in M’diq Bay clearly showed the presence of an acute physiological stress response in both sub-adults and adults. This stress response was quantified using hematological indicators such as cortisol, glucose, lactate and total protein. The results of the present study also showed the existence of a specific response to the age, the individual status and evolution over time of each stressor in *D. labrax*. The use of anti-stress additives offers interesting perspectives to alleviate this problem and further develop the productivity of this fish species.

## Figures and Tables

**Table 1:** Morphometric description of the two studied age groups of the European sea bass (subadults and adult fish); two-way ANOVA and Student’s *t*-test (***: *p* < 0.001).

European Seabass	Age	Days of Stay at the Farm	Weight (g)	Length (cm)
**Subadults** (N = 12)	504 days	364 days (1 year)	125.88 ± 21.33 (87~153)	22.83 ± 1.39 (20~25)
**Adults** (N = 12)	878 days	646 days (1 year and 8 months)	338.88 ± 13.95 *** (312~363)	31.4 ± 0.76 *** (30~33)

**Table 2:** Organosomatic indices of the studied sub-adult and adult European sea bass (VSI: Viserosomatic Index, HSI: Hepatosomatic Index, ISI: Intestine somatic Index, SSI: Spleen somatic Index, GSI: Gonadosomatic Index); two-way ANOVA and Student’s *t*-test (* *p* < 0.05; *** *p* < 0.001).

European Seabass	Organosomatic Indices				
	VSI	HSI	ISI	SSI	GSI
**Subadults** (N = 10)	10.69 ± 1.28	1.70 ± 0.35	1.84 ± 0.20	0.05 ± 0.01	__
**Adults** (N = 10)	11.51 ± 1.01 *	1.66 ± 0.13	1.70 ± 0.16	0.12 ± 0.03 ***	0.49± 0.09 ***

**Table 3:** Presentation of the concentrations of blood stress indicators (mean ± SD; Max~Min) obtained in subadult and adult sea bass in farming conditions at the M’diq Bay, in pre- and post-sampling states. Significance thresholds are shown by two-way ANOVA, Kruskal-Wallis test, Levene and Student’s *t*-tests, and Bonferroni’s post-hoc correction (with *: *p* < 0.05, **: *p* < 0.01, ***: *p* < 0.001, NS: *p* > 0.05).

Sampling for Weight-Length Measurement							
European Seabass	Subadult Fish (N = 24)		Adult Fish (N = 24)		*p* Value		
Stress Indicators	Pre-Stress (N = 12)	Post-Stress (N = 12)	Pre-Stress (N = 12)	Post-Stress (N = 12)	‘Age’	‘Stress -State’	‘Age & Stress-State’
Cortisolemia (ng/mL)	115.74 ± 22.32 (88.60~151.60)	264.54 ± 50.34 *** (173.11~341.23)	187.34 ± 46.20 (122.72~246.86)	320.70 ± 72.66 *** (193.87~405.00)	<0.001	<0.001	NS
Glycemia (mmol/L)	4.99 ± 1.93 (3.05~9.27)	9.53 ± 3.25 *** (3.94~14.26)	7.60 ± 3.20 (3.77~13.04)	14.05 ± 4.67 ** (5.99~19.92)	<0.01	<0.001	NS
Lactatemia (mmol/L)	9.12 ± 4.55 (3.00~16.65)	13.61 ± 3.56 * (6.88~19.43)	10.35 ± 5.38 (1.00~20.87)	17.65 ± 6.83 ** (5.66~29.42)	NS	<0.001	NS
Cholesterol (mmol/L)	5.83 ± 1.52 (3.88~10.17)	5.58 ± 0.82 (3.75~6.68)	4.58 ± 0.71 (3.26~5.38)	5.47 ± 1.39 (3.34~7.84)	NS	NS	NS
Total protein (g/dL)	6.25 ± 1.10 (4.50~8.10)	4.98 ± 1.25 * (3.00~7.80)	6.92 ± 0.65 (5.40~7.50)	5.66 ± 0.81 *** (4.20~7.50)	<0.05	<0.001	NS
Hematocrit (%)	40.91 ± 9.46 (20.00~54.54)	40.87 ± 13.92 (16.67~60.53)	56.27 ± 11.09 (42.85~77.78)	44.12 ± 14.32 * (25.00~70.33)	<0.05	NS	NS

**Table 4:** Linear correlation (correlation coefficient and significance threshold) between the evolution of blood biomarkers in the farmed D. labrax at M’diq Bay (with ***: *p* < 0.001; **: *p* < 0.01; *: *p* < 0.05; NS: *p* > 0.05).

European Seabass Blood Parameters (N = 48 Fish)						
	Cortisolemia (ng/mL)	Glycemia (mmol/L)	Lactatemia (mmol/L)	Total Cholesterol (mmol/L)	Total Protein (g/dL)	Hematocrit (%)
**Cortisolemia** (ng/mL)	−	0.85 ***	0.65 ***	−0.08 ^NS^	−0.25 *	− 0.03 ^NS^
**Glycemia** (mmol/L)		−	0.56 ***	−0.12 ^NS^	−0.07 ^NS^	0.04 ^NS^
**Lactatemia** (mmol/L)			−	− 0.12 ^NS^	− 0.17 ^NS^	− 0.13 ^NS^
**Total Cholesterol** (mmol/L)				−	− 0.06 ^NS^	− 0.24 ^NS^
**Total protein** (g/dL)					−	0.41 **
**Hematocrit** (%)						−

## Data Availability

Informed consent was obtained from all subjects involved in the study.
